# Application of *in silico* Platform for the Development and Optimization of Fully Bioresorbable Vascular Scaffold Designs

**DOI:** 10.3389/fmedt.2021.724062

**Published:** 2021-10-14

**Authors:** Miljan Milosevic, Milos Anic, Dalibor Nikolic, Vladimir Geroski, Bogdan Milicevic, Milos Kojic, Nenad Filipovic

**Affiliations:** ^1^Bioengineering Research and Development Center, BioIRC, Kragujevac, Serbia; ^2^Institute for Information Technologies, University of Kragujevac, Kragujevac, Serbia; ^3^Faculty of Information Technologies, Belgrade Metropolitan University, Belgrade, Serbia; ^4^Faculty of Engineering, University of Kragujevac, Kragujevac, Serbia; ^5^Department of Nanomedicine, Houston Methodist Research Institute, Houston, TX, United States; ^6^Serbian Academy of Sciences and Arts, Belgrade, Serbia

**Keywords:** *in vitro* mechanical test, vascular scaffold, bioresorbable PLLA stent, design and optimization, finite element analysis

## Abstract

Bioresorbable vascular scaffolds (BVS), made either from polymers or from metals, are promising materials for treating coronary artery disease through the processes of percutaneous transluminal coronary angioplasty. Despite the opinion that bioresorbable polymers are more promising for coronary stents, their long-term advantages over metallic alloys have not yet been demonstrated. The development of new polymer-based BVS or optimization of the existing ones requires engineers to perform many very expensive mechanical tests to identify optimal structural geometry and material characteristics. *in silico* mechanical testing opens the possibility for a fast and low-cost process of analysis of all the mechanical characteristics and also provides the possibility to compare two or more competing designs. In this study, we used a recently introduced material model of poly-l-lactic acid (PLLA) fully bioresorbable vascular scaffold and recently empowered numerical InSilc platform to perform *in silico* mechanicals tests of two different stent designs with different material and geometrical characteristics. The result of inflation, radial compression, three-point bending, and two-plate crush tests shows that numerical procedures with true experimental constitutive relationships could provide reliable conclusions and a significant contribution to the optimization and design of bioresorbable polymer-based stents.

## Introduction

Coronary artery disease (CAD) is a serious condition caused by the buildup of plaque in the coronary arteries. It affects millions of people around the world and is one of the leading causes of death globally. CAD can be treated with percutaneous transluminal coronary angioplasty (PTCA), which is a minimally invasive method that has revolutionized the treatment of CAD in the last 20 years (1). PTCA procedure, also called percutaneous coronary intervention, opens blocked, or stenosed coronary arteries allowing unobstructed blood flow to the heart tissue (2). A balloon catheter is inserted into the radial or femoral artery along with a stent steered to the point of interest, and finally dilated to compress the atherosclerotic plaque against the arterial wall. The balloon is then deflated and removed, while the stent is left to maintain passability (3) and prevent acute recoil and vessel closure in the first few months after intervention (1). Despite the high clinical success and a relatively low complication risk, PTCA is not an ideal method and has certain limitations such as potential inflammation, late-stent thrombosis, neoatherosclerosis, and restenosis (4–6).

Bioresorbable vascular scaffolds (BVS) were developed to overcome the limitations and disadvantages of PTCA (7, 8). BVS refers to a bioresorbable or biodegradable stent that serves as a temporal scaffold in the first 6–12 months after PTCA. During that period, the artery is fully remodeled and mechanical support of the artery is no longer needed; therefore, the stent degrades and is absorbed over time, typically in 12–24 months after PTCA. Scaffolds are ultimately reabsorbed by the body of the patient after 36 months (9) and excreted as harmless metabolic waste. Among a small range of commercially available BVS types, results of *in vitro* clinical investigation for Absorb (Abbott Lab) stent prototype showed appropriate mechanical support (e.g., radial strength) of the vessel wall after PTCA and degradation resistance (10, 11).

There are currently two groups of materials that can be considered as a material of choice that can be used for BVS production, namely, bioresorbable polymers and biocorrosive metal alloys. Experimental studies have shown that mechanical properties (e.g., stiffness) of bioresorbable polymers are substantially lower than those of permanent or metal alloy stents. This could lead to a significant recoil, early or late (12–14), which may result in many dangerous complications. On the other hand, biocorrosive metal alloys have better radial strength but unpredictable degradation rate, which increases the risk for thrombosis and restenosis (15). Some scientists have concluded that the use of bioresorbable polymers in biomedical applications provides many benefits, especially for coronary stents (16). However, their long-term advantages over metallic drug-eluting stents have not yet been presented (17).

A large number of studies have been performed in the past in order the effects of stent design and stent geometrical parameters on the recoil and stent deployment process. It was also shown in Pauck and Reddy (18) that the stiffness of the polymers is ~100 times lower than that of stainless steel materials, which will result in higher recoil after implantation. One of the studies (19) was performed on the Palmaz–Schatz stent and the results were compared to the Carbostent and Multi-Link Tetra stent (20). The comparison showed that stent geometry and parameters, such as artery surface ratio and stent strut thickness, had a notable impact on the stent deployment process, rates of radial and longitudinal recoil, and rates of dog-boning. Therefore, there is a need for new polymeric stent designs and geometries to compensate for this lower material stiffness (21).

Standard mechanical tests are required for the stents produced for deployment within coronary arteries according to ISO standards. The development of a stent design that successfully passes all experimental tests is time-consuming, a difficult, and an expensive process, which consists of several stages and requires many cycles of mechanical testing and redesigning of the basic model. On the other hand, *in silico* mechanical tests could reduce the cost and the number of necessary real mechanical tests. An appropriate loading scenario within *in silico* tests allow manufacturers to compare the axial and radial conformability of their stent design with competitors on the market or with their own older designs. Therefore, computational numerical methods have a great potential to be used as a mighty and robust tool in the evaluation of stent performance and optimization of stent design.

The finite element method (FEM) has proven to be the most powerful computational tool for investigating stent properties and stent design optimization. FEM is a method of choice when dealing with complex geometries, sophisticated non-linear material properties, or cases where material parameters would be impossible to obtain using experimental tests. It is also important to improve the current methodology used for BVS (22) since the major problem is to accurately describe the mechanical behavior of bioresorbable polymers (4, 23). It was shown that FEM can be efficiently used for the prediction of structure degradation during cyclic loading (19), or the construction of new material models (24–28). In Szymonowicz et al. (15), the authors presented an advanced Bergstrom–Eswaran material model, based on hyperelasticity and visco-plastic effects. A similar study was described in Lin et al. (29), but this approach requires a significant number of material constants. Another approach was provided by Schiavone et al. (30), where material stiffening was implemented as a function of plastic strain. However, the main disadvantage of this method is the lack of a strain rate or anisotropic representation (31). Recently, a group of authors (1, 16) adopted the general elastic-plastic material while taking into account the strain rate modeling strategy and kinematic/isotropic hardening. Therefore, additional *in vitro* studies have to be performed in this field to appropriately characterize the performance of bioresorbable polymeric scaffolds.

One step forward in that direction was made in the study by Filipovic et al. (32) where the authors developed a finite element (FE) model for the investigation of partial and full BVS manufactured by Boston Scientific Limited (Galway, Ireland) (33). For partially bioresorbable SYNERGY™ BP (Bioabsorbable Polymer) Everolimus-Eluting Platinum Chromium Coronary Stent made from the platinum–chromium alloy (Pt–Cr), we used an elasto-plastic material model and material parameters from O'Brien et al. (34). For the prototype polymeric fully bioresorbable stent made from PLLA, we introduced a new material model based on experimentally obtained uniaxial tensile stretch–stress relations. The analysis was performed at three different strain rates (0.001, 0.01, and 0.1 s^−1^) and three different temperatures (25, 37, and 48°C). A comparison of the results between the simulation and real experiments for the inflation, radial compression, and crush resistance tests were given with the coefficient of determination and correlation coefficient. In most cases, the strong matching between *in vitro* and *in silico* results was shown, for either partially or fully bioresorbable stents. It was proof that numerical simulation can be used instead of standard *in vitro* mechanical experiments with BVS. Our motivation for “direct” use of measured relationships was to include the constitutive curves directly into the FE models. The advantage of this approach is its relatively simple implementation with a satisfactory representation for bioresorbable polymers. Moreover, while using this approach the problem with the lack of a strain rate mentioned in Szewczenko et al. (31) does not emerge, since we used information directly from experimental curves. Therefore, this study was a step further in providing a new way of *in vivo* and *in vitro* characterization of stent performance, since there is no sufficient data on the use of biodegradable materials for implantable stents.

In this study, we compared and analyzed the impact of strut thickness on mechanical characteristics of AB–BVS scaffold, and also the impact of additional pocket holes (slots) in stent geometry on mechanical characteristics of Renuvia–PLLA stent. The comparison and analysis were performed by using an FE numerical simulation and a recently introduced material model (32) for the prototype of the polymeric fully bioresorbable stent. The numerical results and corresponding analysis are provided for each of the stent designs, and for four different tests: radial compression, inflation, three-point bending, and two-plate crush resistance.

## Materials and Methods

### Stent Designs

An *in silico* investigation was performed for two stent prototypes provided by different manufacturers. Characteristics of the stents are provided in Table 1.

**Table 1:** Characteristics of the stent.

**Sent name**	**AB-BVS**	**AB-BVS thinner**	**PLLA prot**	**PLLA prot-slots**
Outer radius [mm]	1.65	1.62	1.62	1.62
Inner radius [mm]	1.49	1.49	1.5	1.5
Length [mm]	12.18	12.18	15.68	15.68
No of hexahedral FE elements	46,728	46,728	49,488	40,908

**Figure 1:**
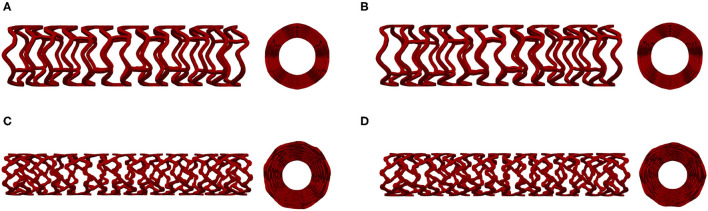
Stent geometry: AB-BVS **(A)**, AB-BVS-thinner **(B)**, PLLA prot **(C)**, PLLA prot-slots **(D)**.

Figure 1 shows the geometry of all stents used in our *in silico* investigation. The first stent design called absorb (AB-BVS) is a preproduction stent prototype and was supplied by Abbott. For this stent, there are two different geometries (AB–BVS and AB–BVS-thinner) that differ in the thickness of the stent strut (Figures 1A,B). As can be seen in Table 1, the thickness of AV–BVS is 160 μm whereas the thickness of AB–BVS-thinner is 130 μm. Geometry for Renuvia–PLLA prototype stent was supplied by Boston Scientific Limited (Galway, Ireland) (33). Two different geometries were compared (Figures 1C,D), namely the original Renuvia–PLLA geometry and the preproduction prototype with additional pocket slots. The length of the Renuvia–PLLA prototype stent is 15.68 mm, the internal diameter is 3 mm, the strut width is 184 μm, and the strut thickness is 115 μm.

An FE mesh of the model was then generated from the input geometry files. The number of FE elements for each of the models is presented in Table 1.

All of the stent designs were supposed to be made from poly-l-lactic acid (PLLA) material. PLLA is an elasto-visco-plastic polymer, the mechanical deformations of which are the functions of both strain rate and temperature. For that reason, we used the recently introduced material model for a polymeric fully bioresorbable stent.

### Computational Procedure

Computations were performed using our in-house FE code called PAK [Program zaAnalizuKonstrukcija, (35)]. PAK is able to solve all types of non-linearities, such as large deformations, geometrical non-linearities, and contact problems. We used displacement formulation, i.e., nodal variables are displacement, convenient to solid mechanics problems, whereas stresses in solids were calculated from strains or stretches. The balanced equation of a FE can be written in the form as follows given by Kojic et al. (36):


(1) $$\begin{array}{l} \left(\frac{1}{\Delta t^{2}}\text{M}+\text{K}\right)\Delta {\text{U}}^{\left(i\right)}=\\ {\text{F}}^{ext}-{\text{F}}^{\mathrm{int}\left(i-1\right)}-\frac{1}{\Delta t^{2}}\text{M}\left({\text{U}}^{\left(i-1\right)}-{\text{U}}^{t}\right) \end{array}$$

where the mass and stiffness matrices **M** and **K** have a standard form (36), and **U** and **U**^*t*^ are nodal velocities at the current (or previous) iteration and at the start of a time step, respectively; **F**^*ext*^ and **F**^*int*^ are external and internal nodal forces, respectively; and nodal variables are one-dimensional arrays.

### Material Model of the BVS Prototype of a Polymeric Bioresorbable Stent

**Figure 2:**
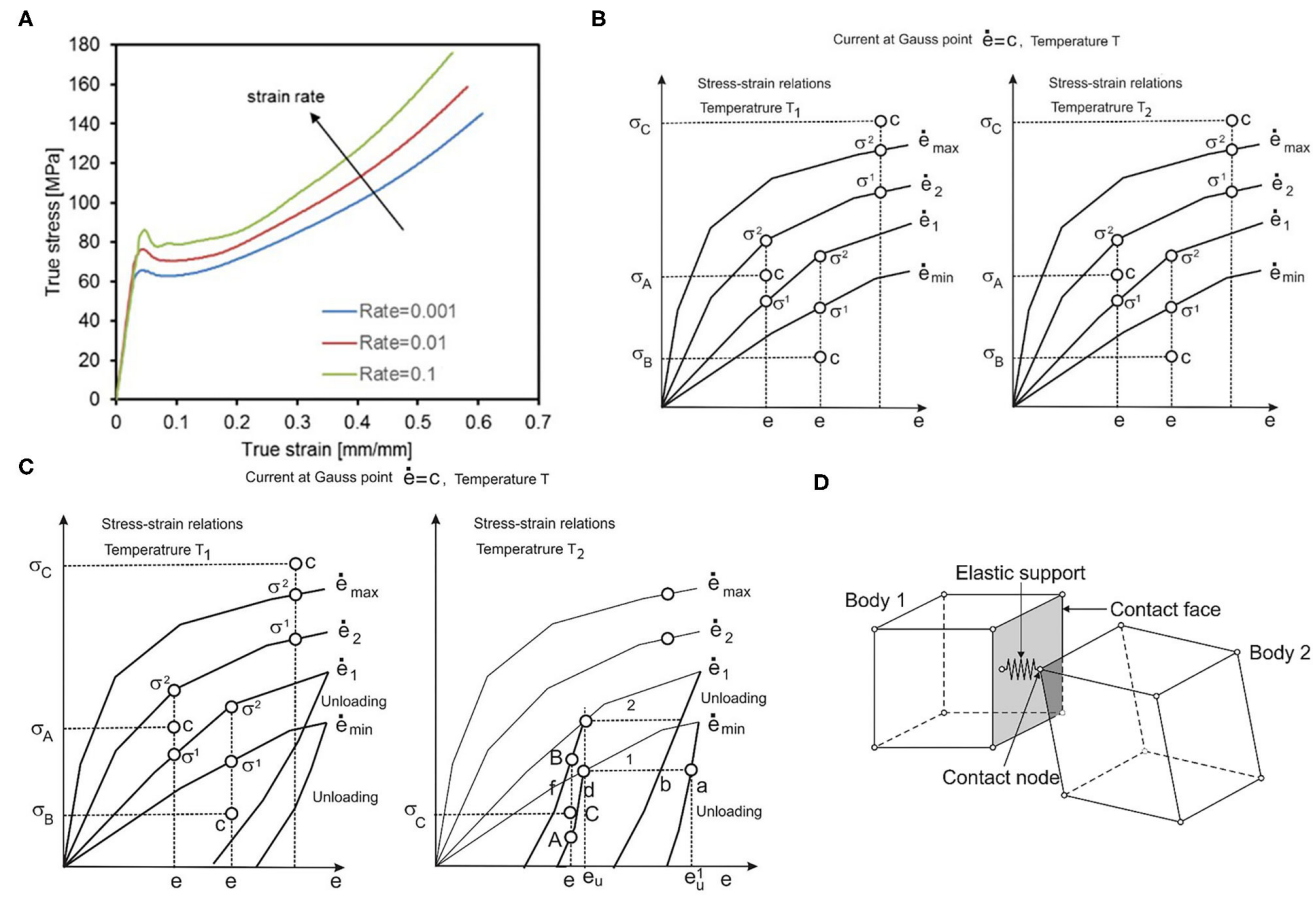
**(A)** Average tensile test results provided by BSL and strain rates, **(B)** Uniaxial stress-strain curves for different strain rates and one temperature, **(C)** with unloading curves, one unloading curve corresponds to each loading curve. **(D)** Interaction between solid bodies, two bodies in contact, and elastic support placed in contact point.

All of the stent designs were supposed to be made from PLLA material. PLLA is an elasto-visco-plastic polymer, the mechanical deformations of which are the function of both strain rate and temperature. For that reason, we used the recently introduced material model for the polymeric bioresorbable stent, presented in Filipovic et al. (32). This material model is based on experimentally detected stretch vs. stress curves. Experimental curves were provided for different temperatures (25°C, 37°C, and 48°C) and different strain rates (0.001, 0.01, and 0.1 s^−1^). Experimental curves for a temperature of *T* = 25°C and different strain rates are shown in Figure 2A. As can be seen in Figure 2A, there is an initial elastic zone preceded by a yield point and plastic behavior. Input in this material model can be any multilinear curve provided by manufacturers and investigators, which is the main advantage of this approach in comparison with commercial FE packages and codes.

To be used in a FE numerical simulation, we need to make a representation of the 3D stress-strain state by using uniaxial experimental curves. All derivations with the FE model are based on the principle of equivalence of virtual work in 1D and 3D stress-strain conditions. We start with the equations for equivalent stress $\overset{-}{\sigma }$ and strain ē:


(2) $$\begin{array}{l} \overset{-}{\sigma }=\{\frac{1}{2}[{\left(\sigma_{11}-\sigma_{22}\right)}^{2}+{\left(\sigma_{22}-\sigma_{33}\right)}^{2}+{\left(\sigma_{33}-\sigma_{11}\right)}^{2}+\\ \;6\left(\sigma_{12}^{2}+\sigma_{23}^{2}+\sigma_{31}^{2}\right)]\} \end{array}$$

(3) $$\begin{array}{l} ē={\left[\frac{2}{3}\left(e_{11}^{2}+e_{22}^{2}+e_{33}^{2}\right)+\frac{1}{3}\left(\gamma_{12}^{2}+\gamma_{23}^{2}+\gamma_{31}^{2}\right)\right]}^{1/2} \end{array}$$

which were taken from Kojic and Bathe (37), where σ_*ij*_ are stresses; *e*_*ij*_ are strains, and γ_*ij*_ are engineering strains. Values for equivalent stress and equivalent strain are calculated for each Gauss integration point of the FE, according to experimental true strain vs. true stress curves. Uniaxial stress-strain curves for different strain rates at temperature T_1_ are schematically shown in Figure 2B. According to these curves, using interpolation procedure, we calculate equivalent stress for current strain, which is further used in our model. First, we interpolate equivalent stress for current strain, current strain rate, and current temperature (32):


(4) $$\begin{array}{l} \sigma_{T1}={\left(\sigma_{1}+\frac{\sigma -\sigma_{1}}{\sigma_{2}-\sigma_{1}}\right)}_{T1},\;\sigma_{T2}={\left(\sigma_{1}+\frac{\sigma -\sigma_{1}}{\sigma_{2}-\sigma_{1}}\right)}_{T2} \end{array}$$

where it is assumed that curves 1 and 2 correspond to strain rates and ė_2_, and temperatures *T*_1_ and *T*_2_. It is additionally assumed that ė_1_ < ė < ė_2_, σ_1_ < σ < σ_2_ and T_1_ < *T* < *T*_2_. We finally interpolate for temperature:


(5) $$\begin{array}{l} \sigma_{T}=\sigma_{T1}+\frac{T-T_{1}}{T_{2}-T_{1}}\left(\sigma_{T2}-\sigma_{T1}\right) \end{array}$$

If ė is less than ė_min_, we use the equation:


(6) $$\begin{array}{l} \sigma_{T1}=\frac{ė}{{ė}_{\min}}\sigma_{1}\text{ or }\sigma_{T2}=\frac{ė}{{ė}_{\min}}\sigma_{2} \end{array}$$

and in the case when ė is greater than ė_max_, we use the equation:


(7) $$\begin{array}{l} \sigma_{T1}=\sigma_{\max}+\frac{ė-{ė}_{\max}}{{ė}_{\max}-{ė}_{\max-1}}\left(\sigma_{\max}-\sigma_{\max-1}\right) \end{array}$$

The computational steps for the model using experimental curves are divided into three phases: loading, unloading, and reloading (32). In the loading phase, based on the current equivalent strain and current equivalent strain rate, we calculate the final stress and factorize the tangent matrix. Thus, for the current time step and iteration, we have the following steps:

- Calculate equivalent stress ${\overset{-}{\sigma }}^{\left(i-1\right)}$ and tangent constitutive matrix $C_{ij}^{E\left(i-1\right)}$, for the current equivalent strain ē^(*i*−1)^ and equivalent strain rate ${\dot{ē}}^{\left(i-1\right)}$, using a tangent elastic module $E_{T}^{\left(i-1\;\right)}$.
- Calculate stress increments and stresses from the given curves as $\Delta \sigma_{k}^{\left(i\right)}=C_{kj}^{E\left(i-1\right)}\Delta e_{j}^{\left(i-1\right)}$ and $\sigma_{k}^{\left(i\right)}=\sigma_{k}^{t}+C_{kj}^{E\left(i-1\right)}\Delta e_{j}^{\left(i-1\right)}$, respectively.
- Calculate ${\overset{-}{\sigma }}_{new}^{\left(i-1\right)}$from stresses and evaluate the stress ratio $r_{stress}=\frac{{\overset{-}{\sigma }}^{\left(i-1\right)}}{{\overset{-}{\sigma }}_{new}^{\left(i-1\;\right)}}$
- Calculate final stresses as $\sigma_{k\left(final\right)}^{\left(i\right)}=r_{stress}\sigma_{k}^{\left(i\;\right)}$
- Factorize the tangent matrix as $C_{\left(final\right)kj}^{E\left(i-1\right)}=r_{stress}C_{kj}^{E\left(i-1\;\right)}$

Additionally, we can calculate elastic and plastic strains using $\Delta e_{i}^{E}={\left(C_{ij}^{E}\right)}^{-1}\;\left(\sigma_{i}\;-\;\sigma_{j}^{t}\right)$ and $\Delta e_{i}^{P}=\Delta e_{i}-\Delta e_{i}^{E}$, respectively.

Then, in the unloading phase (Figure 2C), we execute the interpolation procedure and find stress as the function of the unloading strain. The interpolation is performed between curves for different strain rates and different temperatures, in a similar fashion as for effective stress calculation in the loading phase. In the final stage, i.e., reloading, we repeat the loading phase but from the point where the unloading phase ended.

### Implementation of Non-Linear Contact Problem

Within corresponding tests presented in this work, stents were either compressed by cylinder, deformed by parallel plates, or axially compressed in a localized point. Therefore, there was a need to implement a methodology for interaction between outer surfaces of a stent and corresponding moving boundaries, with prescribed loads or displacements that are used to perform stent compression or bending. The methodology implemented in our FE code PAK is based on the mechanism of interactions between two bodies [(38–42)], which is presented in Figure 2D, and non-linear contact problem presented in Isailovic and Filipovic (43). Following the procedure presented in Isailovic and Filipovic (43), each time when a node of moving boundary enters the FE of a stent through the outer surface of the stent, we generate a 1D elastic support element that acts like a spring and tends to separate the two moving bodies. This new elastic 1D support element is added to the system of linear equations, for the current time step of FE simulation. The procedure is repeated for each succeeding time step, or timeframe, until the end of the numerical simulation. To provide a symmetric action-reaction response, this procedure has to be performed for both domains in contact.

### Verification and Validation of *in silico* Models

Detailed verification of the material model used in the FE PAK solver (35) was explained in Filipovic et al. (32). Material model input data is based on experimental results provided by Boston Scientific Limited. By analyzing and comparing *in vitro* and *in silico* diameter–pressure and diameter–load curves in several standard tests, the authors concluded that the performed simulations mimicked the real tests with very high precision (32). The goal of this research was, therefore, to present capabilities of the validated numerical model to simulate the mechanical behavior of preproduction stent prototypes and to eventually in the future avoid or reduce the number of real and expensive mechanical tests.

According to the recently introduced ASME V&V 40 standard document, there is a need to provide proof of the reliability of a numerical model in the area of *in silico* trials (44). In terms of the ASME V&V 40 document, authors of any computational modeling approach in the area of medical devices, among all, has to: (i) define the question of interest (QOI), which is the specific question, decision, or concern that is being addressed, (ii) to present the credibility of the model to a specific context of use (COU), (iii) and to present and discuss risks associated with the model (44). These aspects are crucial as they affect the acceptance of model errors observed during the validation phase (45). In this context, QOI, COU, and risk assessment for our computational model and facts regarding the reliability of the presented *in silico* simulation are presented in the “Discussion” section.

## Results

Characteristics of stent designs were analyzed through four standard and general *in silico* tests including radial compression (RCI), inflation, crush resistance (two-plate), and three-point bending.

### Radial Compression Test

**Figure 3:**
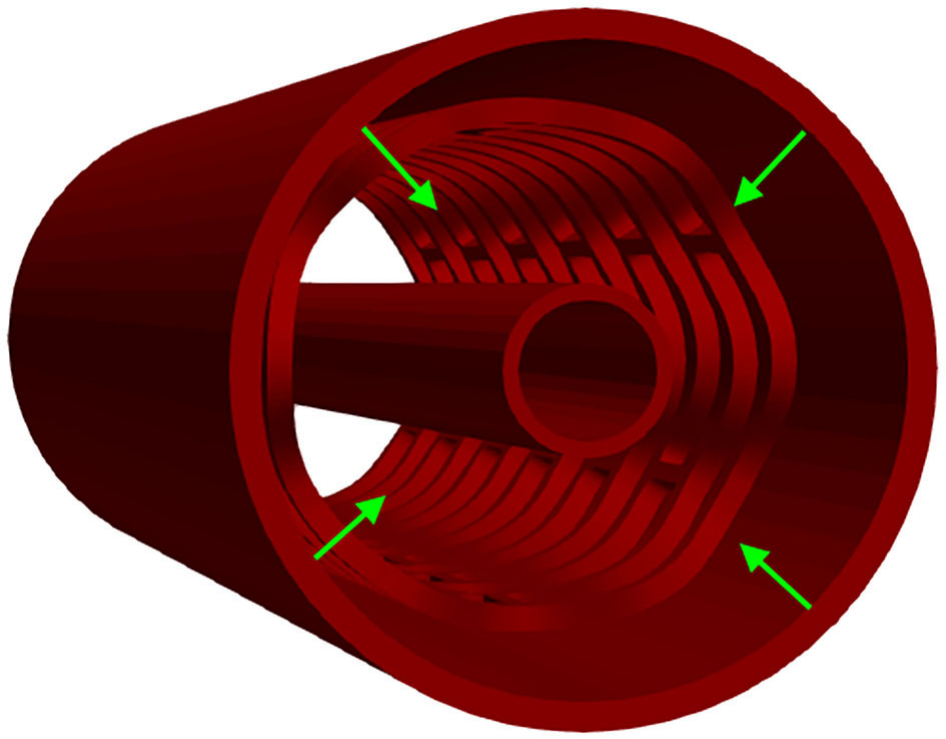
Boundary conditions of a radial compression test.

The *in vitro* experiment used circumferentially uniform radial load to determine the load/deformation characteristics of the stent. The central axis of the stent, in this simulation, was taken to be the *Z*-axis. The boundary condition for nodes at one side of the stent at *z* = *Z*_min_ was *u*_*Z*_ = 0. The stent was compressed using a uniform rate of compression, whereas the non-linear interaction was set between the outer surface of the stent and the inner surface of the cylinder (32). The outer surface of the cylinder was loaded by prescribed radial displacement with the purpose of simulating the “Mylar” loop crimping device. The goal of the *in silico* test was to compress the stent down to the radius of 1 mm to determine the effect of strut thickness and slots on the characteristics of the stent. This was done with the use of cylinders (outer and inner) where the outer cylinder was compressed to simulate a realistic test. The boundary conditions mimicking the test (32) are presented in Figure 3.

**Figure 4:**
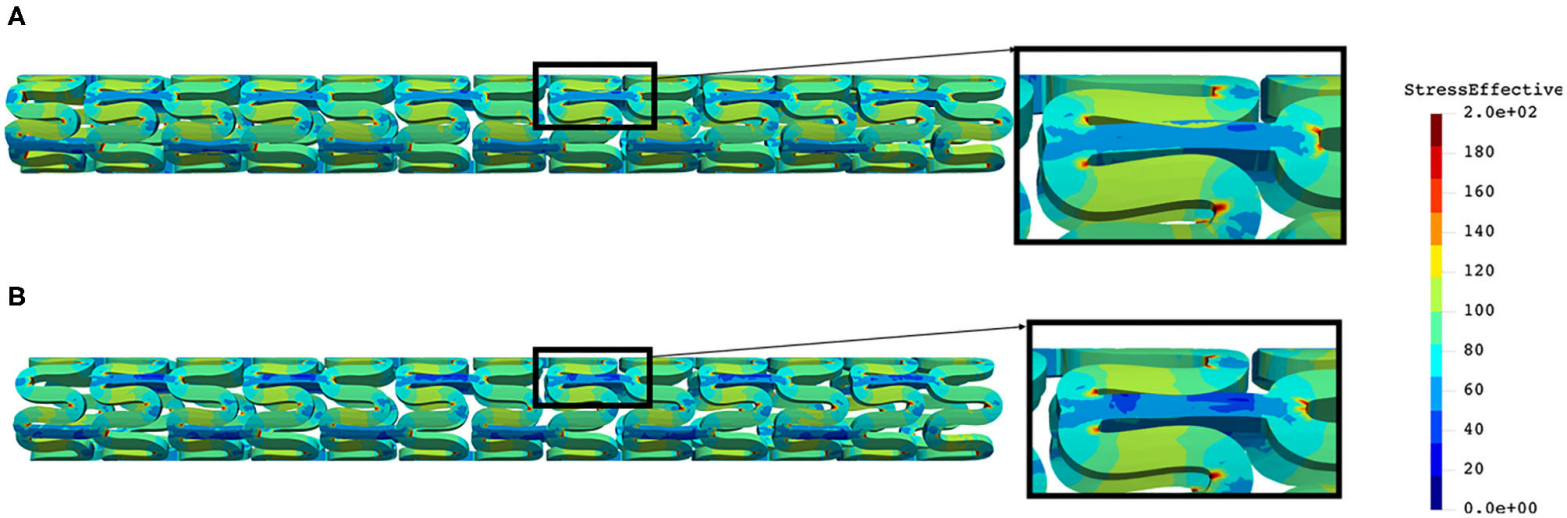
Effective stress distribution on AB–BVS **(A)** and AB–BVS thinner **(B)** stents for radial compression test.

**Figure 5:**
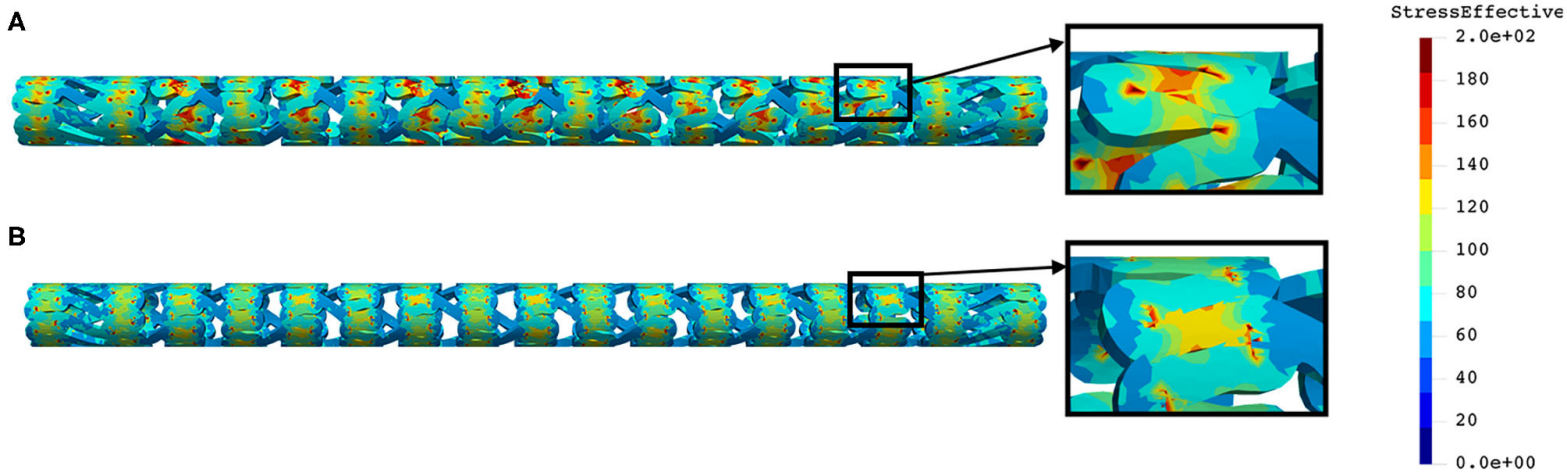
Effective stress distribution on PLLA prot **(A)** and PLLA prot-slots **(B)** stents for radial compression test.

The distribution of effective stress of the radial compression test for AB–BVS and AB–BVS-thinner stents are shown in Figures 4A,B, respectively, whereas the effective stress distribution for Renuvia–PLLA prot and PLLA prot-slots stents is shown in Figures 5A,B, respectively.

As can be observed from Figure 4, the lower thickness of the strut causes higher concentrations of stress on struts. However, that concentration is mostly located in the middle of the strut rather than on the connection between the strut and the rings, as is the case on the thicker model (A), where higher concentrations of stress could result in a critical failure.

From Figure 5 we can conclude that additional slots (B) on the model contribute to better effective stress distribution, thus relaxing the structure of the stent regarding stress concentrations.

### Inflation Test

**Figure 6:**
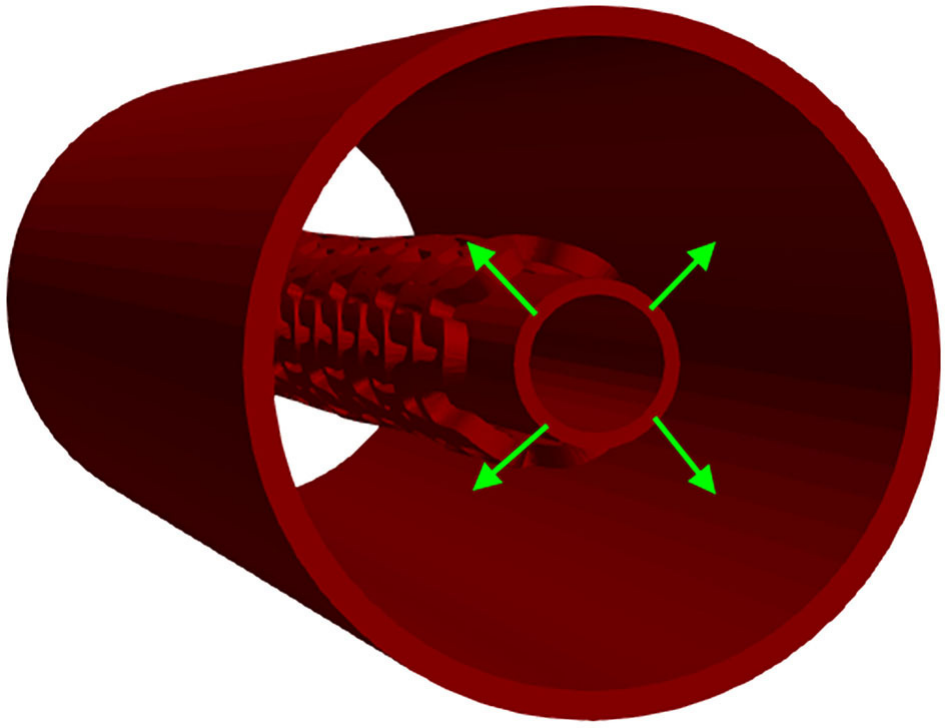
Inflation test. Stent configuration at the end of a radial compression test is taken as input for the inflation test. The stent is then inflated using prescribed pressures applied at the outer surfaces of the inner cylinder.

Although the *in vitro* experiment used the computer-controlled Nexus 500 syringe pump to inflate the stent, with an accuracy of 99.96%, and with the purpose to estimate the required diameter and achieve nominal recommended pressure during balloon inflation, the goal of this *in silico* test was to inflate the stent up to the nominal radius (32) of 3 mm to determine the effect of strut thickness and slots on characteristics of the stent. This was done with the use of cylinders (outer and inner), where the outer cylinder was compressed to simulate a realistic test. The central axis of the stent, in this simulation, was taken to be the *Z*-axis. The boundary condition for nodes at one side of the stent at *z* = *Z*_min_ was *u*_*Z*_ = 0. To provide good numerical stability, we prescribed a very low speed of the inflation process. The stent was inflated by using prescribed pressures applied at the inner surfaces of the balloon (Figure 6). To provide realistic results, results obtained from the radial compression test were taken as an input together with residual stresses. Non-linear body interaction was set between the outer surface of the balloon and the inner surface of the stent.

**Figure 7:**
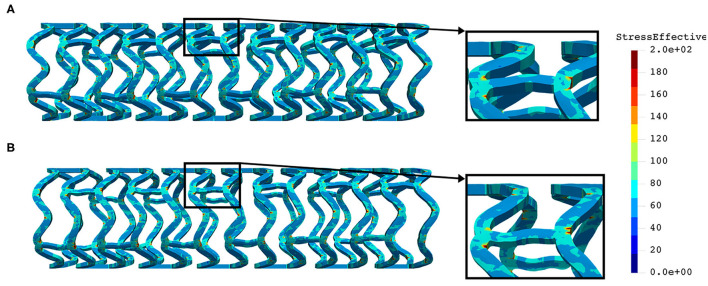
Effective stress distribution on AB–BVS **(A)** and AB–BVS-thinner **(B)** stents for inflation test.

**Figure 8:**
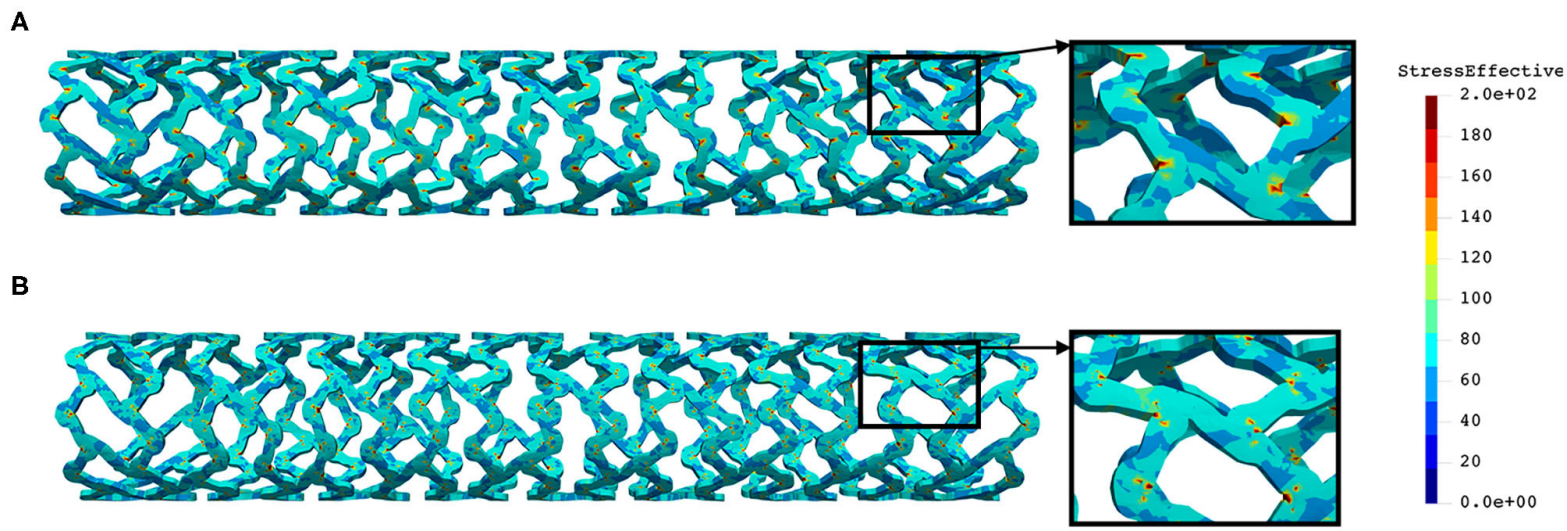
Effective stress distribution on PLLA prot **(A)** and PLLA prot-slots **(B)** stents for inflation test.

The distribution of effective stress from the inflation test of AB–BVS and AB–BVS-thinner stents are shown in Figures 7A,B, respectively, whereas the effective stress distribution for Renuvia–PLLA prot and PLLA prot-slots stents is shown in Figures 8A,B, respectively.

As can be seen in Figure 7, the lower thickness of strut in the inflation test resulted in lower concentrations of stress on struts, thus providing better structural integrity. We can notice in Figure 8 that additional slots on the stent (B) had no significant effect on effective stress distribution during the inflation test.

### Three-Point Bending Test

**Figure 9:**
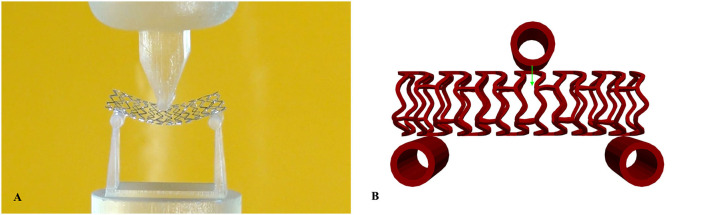
Three-point bending test: experimental set up **(A)**, and boundary conditions **(B)**.

A three-point bending procedure, according to ASTM F2606 standard, was used to quantitatively characterize stent flexibility. The *in vitro* three-point bending test was performed on the axial load testing device AMETEK Brookfield machine with a displacement accuracy of 0.05 mm, equipped with a very precise low force load cell of 9.8 N (resolution of 0.00098N) and a specially designed tool for small size devices. The testing occurred in phosphate-buffered saline at 37°C and pH 7.4, and this is the condition that stands for the clinically relevant environment. The sample was fixed between three-point band fixtures (Figure 9A) and axially loaded by a machine compressing device at a localized point. During the whole process, the machine measured load and displacement.

The central axis of the stent, in this FE simulation, was taken to be the *Z*-axis. The boundary condition for all FE nodes in the XZ coordinate plane was *u*_*Y*_ = 0, and for all stent FE nodes in the *XY* coordinate plane, the boundary condition was *u*_*Z*_ = 0. The central axis of the three rigid body cylinders was taken to be the *X*-axis. An axial force was applied *via* the top cylinder, and the bottom cylinders were fixed. Non-linear body interaction was set between the outer surfaces of the stent and the outer surfaces of the cylinders. Those boundary conditions fully mimicked the real test. The goal of this test was to bend the stent for at least half of its diameter. To provide realistic results, the output of the inflation test was taken as an input together with the residual stresses. The boundary conditions mimicking the test (32) are presented in Figure 9B.

**Figure 10:**
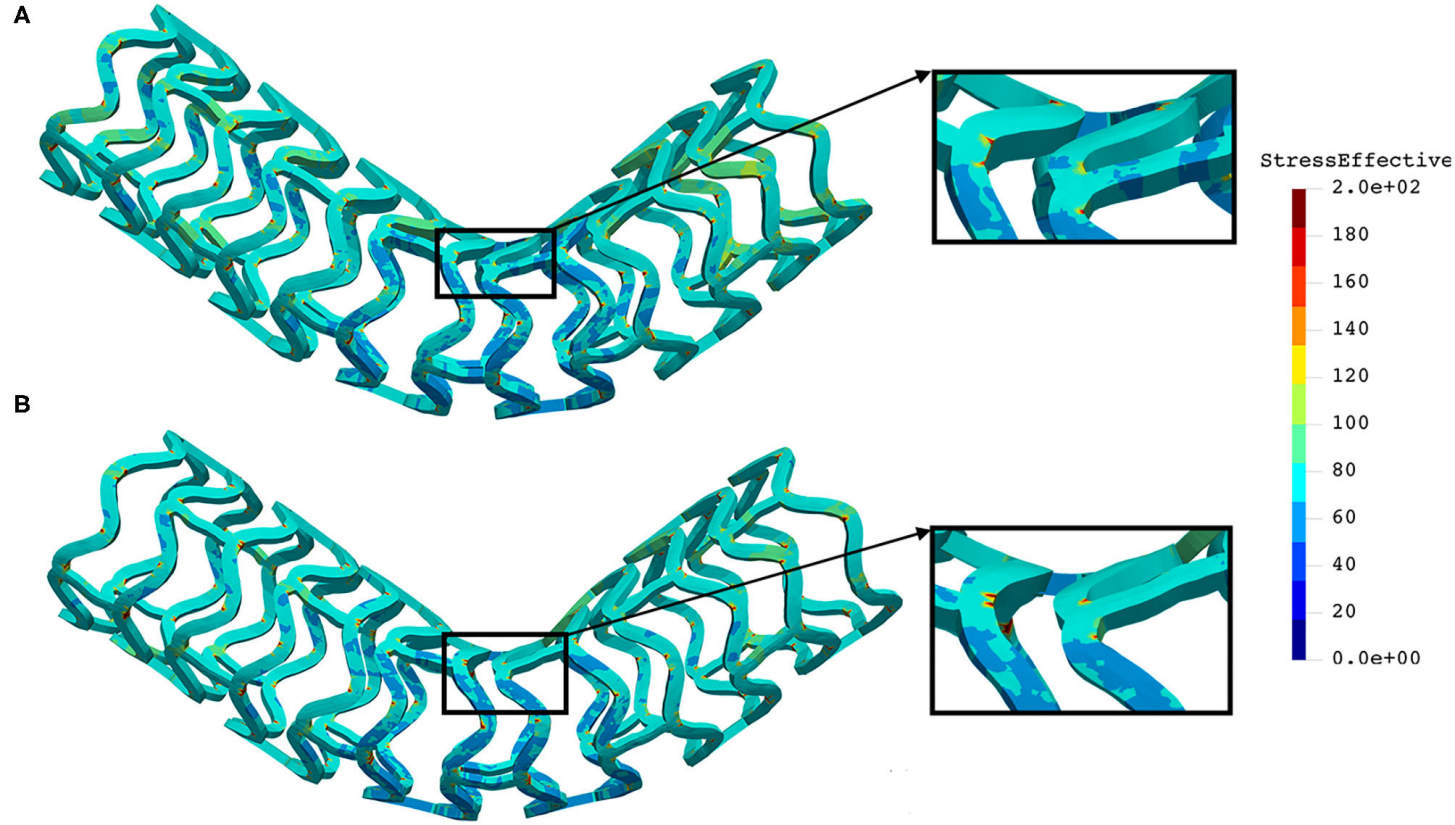
Effective stress distribution on AB–BVS **(A)** and AB–BVS-thinner **(B)** stents for three-point bending test.

**Figure 11:**
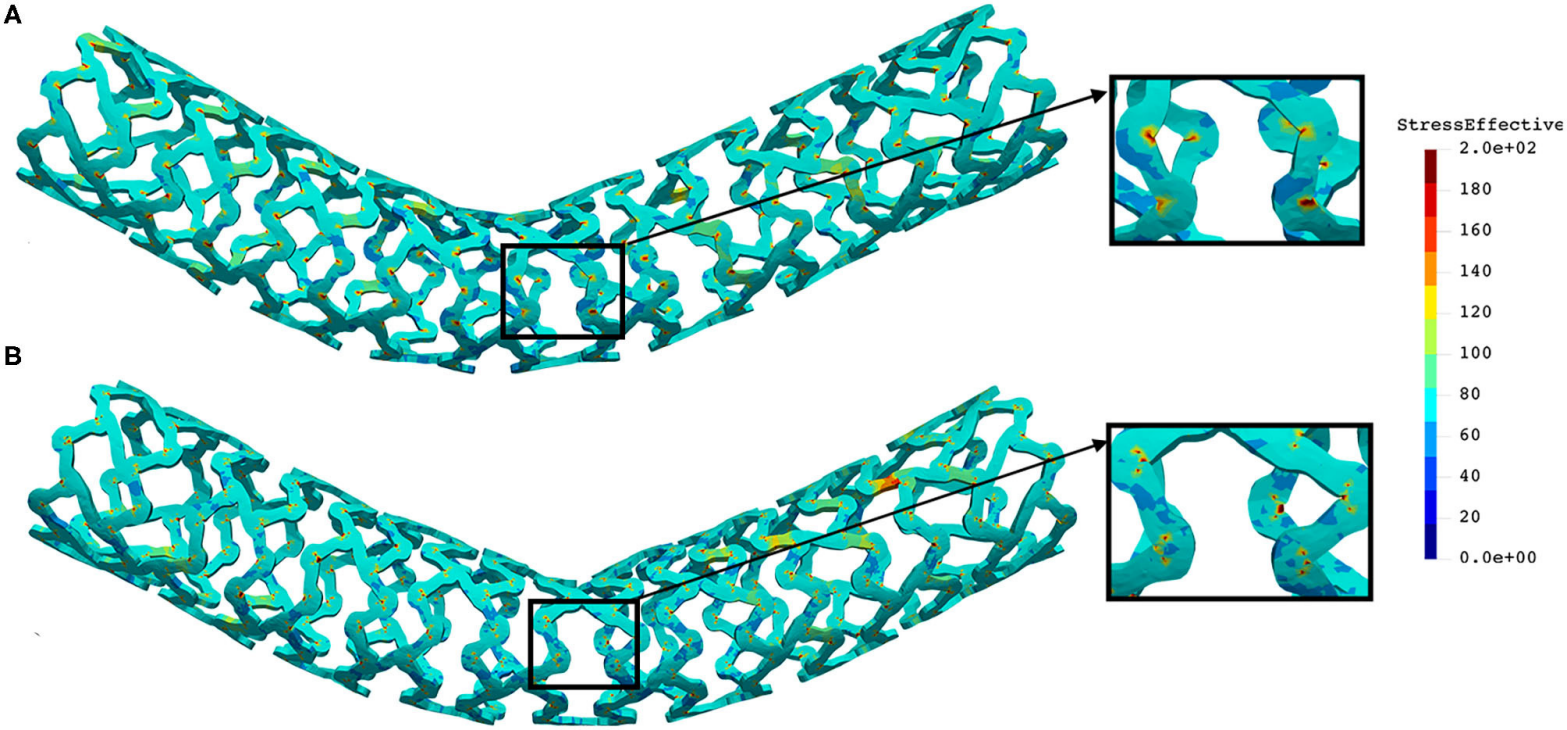
Effective stress distribution on PLLA prot **(A)** and PLLA prot-slots **(B)** stents for three-point bending test.

The distribution of the effective stress from the three-point bending test of AB–BVS and AB–BVS-thinner stents are shown in Figures 10A,B, respectively, whereas the effective stress distribution for Renuvia–PLLA prot and PLLA prot-slots stents is shown in Figures 11A,B, respectively.

As can be observed from Figure 10, the lower thickness of strut in the three-point bending test resulted in lower concentrations of stress on struts, thereby providing better structural integrity. According to the results shown in Figure 11, we can notice that additional slots on the stent (B) had no significant effect on effective stress distribution during the three-point bending test.

### Crush Test/Two-Plate Test

**Figure 12:**
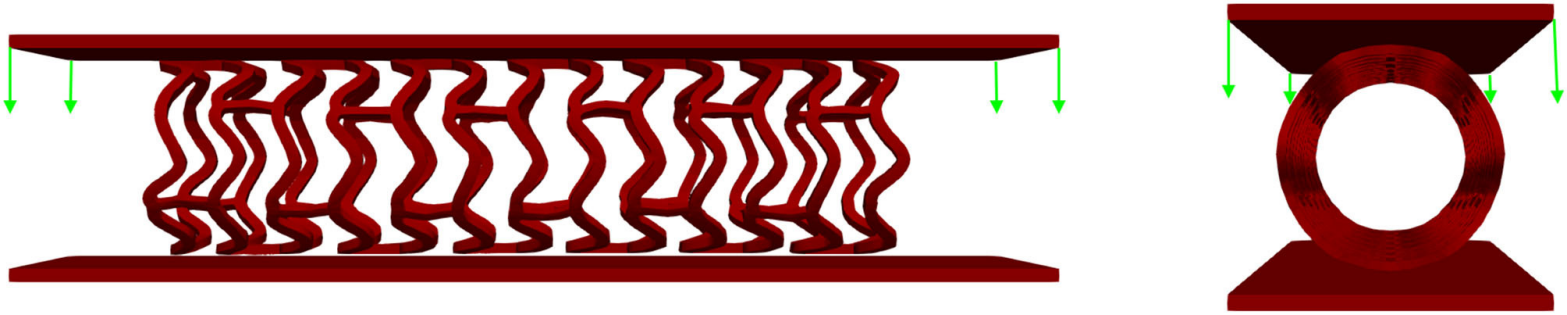
Boundary conditions of crush test. The stent is positioned between two plates: the top plate is loaded by uniform force while the bottom plate is fixed for displacements.

In this *in vitro* test, a stent was positioned between two plates, and a uniform force was applied at one plate whereas the second plate was constrained for movement. The purpose of this test was to estimate the force or load needed to obtain deformation of the stent higher than 50% of its diameter, which is the amount of deflection or bulking relevant for clinical usage. The same amount of deflection was expected to be obtained using *in silico* numerical simulation. The central axis of the stent in this FE simulation was taken to be the *Z*-axis, while the top and bottom plates were placed symmetrically with respect to the Z-axis. The boundary condition for nodes at one end of the stent at *z* = *Z*_min_ was *u*_*Z*_ = 0. The boundary condition for all FE nodes in the *XZ* coordinate plane was *u*_*Y*_ = 0, and for all stent FE nodes in the *YZ* coordinate plane, the boundary condition was *u*_*X*_ = 0. The top plate was loaded by uniform axial force, whereas the bottom plate was constrained for displacements. To provide realistic results, the output of the inflation test was taken as an input together with the residual stresses. Non-linear body interaction was set between the outer surfaces of the stent and surfaces of the plates that were in contact with the stent. The boundary conditions for this crush test (32) are presented in Figure 12.

**Figure 13:**
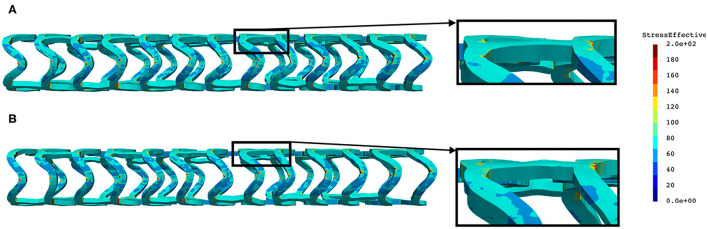
Effective stress distribution on AB-BVS **(A)** and AB-BVS thinner **(B)** stents for crush test.

**Figure 14:**
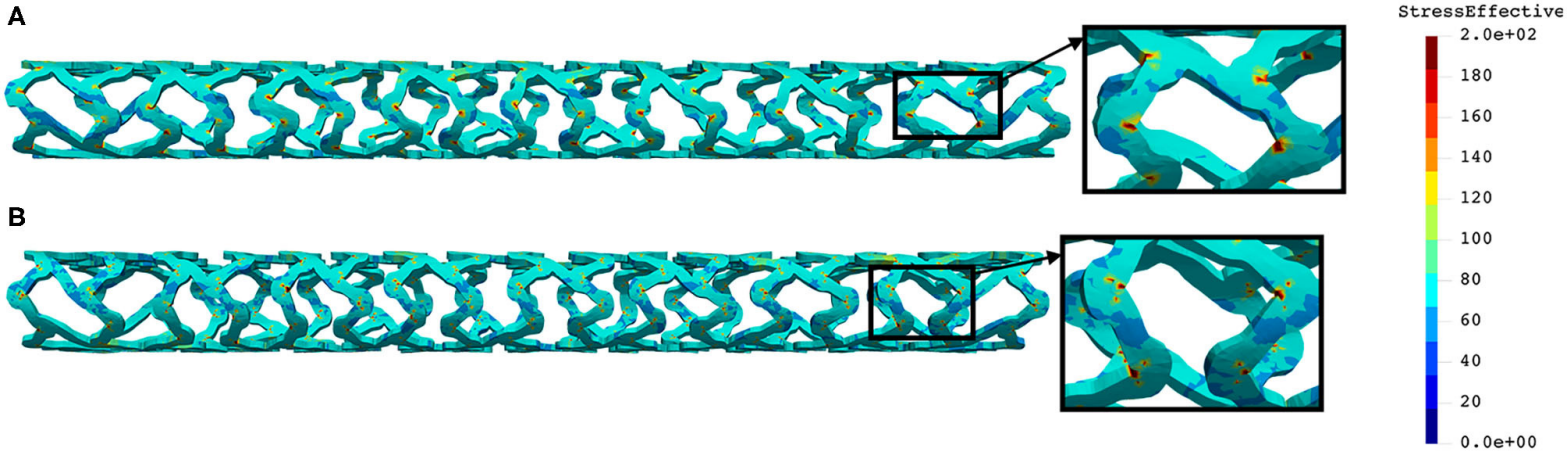
Effective stress distribution on PLLA prot **(A)** and PLLA prot-slots **(B)** stents for crush test.

The distribution of the effective stress from the crush test of AB-BVS and AB–BVS-thinner stents is shown in Figures 13A,B, respectively, whereas the effective stress distribution for Renuvia–PLLA prot and PLLA prot-slots stents is shown in Figures 14A,B, respectively.

As can be observed from Figure 13, the lower thickness of strut in the three-point bending test resulted in lower concentrations of stress on struts, thereby providing better structural integrity. According to the results in Figure 14, we can notice that additional slots on stent (B) had no significant effect on the effective stress distribution during the crush test.

## Discussion

In this study, we presented the abilities of the *in silico* platform for mechanical testing of stents by comparing and analyzing two different polymeric bioresorbable stent designs, namely, AB–BVS and Renuvia–PLLA, with respect to different geometrical characteristics. We chose these types of stents since they are of particular interest for ongoing InSilc project (46), and additionally due to the following reasons. The results for the Renuvia–PLLA stent design were validated using our numerical model (32). Therefore, in this study, the aim was to compare the existing design with a preproduction prototype that has additional pocket holes (slots). On the other hand, for AB–BVS preproduction prototypes, the goal was to provide numerical estimation and to help manufacturers choose among two designs with different strut thicknesses. The polymeric material used for both designs is characterized by low stiffness, strain rate sensitivity, and plastic behavior. Therefore, it presents a challenge to develop a corresponding modeling strategy. The *in silico* simulation platform is based on a recently introduced and validated material model for PLLA, based on experimentally detected true strain–true stress curves, estimated during uniaxial tensile tests for different strain rates and different temperatures. The introduced FE material model showed the ability to be used in running simulations with different stent designs, different geometry and material characteristics, and with the ability to provide reasonable accuracy. It was also shown that input in this material model could be any multilinear curve provided by manufacturers and investigators, which is the major advantage of this approach in comparison to commercial FE packages and codes. The presented FE modeling methodology relies on true experimental constitutive relationships without any parameter fitting and can serve as the basis for practical applications. Additionally, hysteretic characteristics of stent deformation during the unloading phase were included, and it may be of interest to gain an insight into the mechanical characteristics during cyclic stent loading. Our methodology can be improved in the future to include degradation effects to investigate the interaction between a BVS and a coronary artery throughout the degradation process.

We investigated the impact of strut thickness and the impact of additional pocket holes (slots) in stent geometry the field of deformations and the amount of effective stress. Results were presented for radial compression, inflation, three-point bending, and crush resistance/two plates *in vitro* tests. Deformed geometry and residual stresses from the radial compression test were then taken as starting values for other *in vitro* tests. As can be seen from the “Results” section for a radial compression test, the lower thickness of strut of AB–BVS stent caused higher concentrations of stress on struts, which could result in a critical failure. On the contrary, the lower thickness of strut of AB-BVS stent in inflation, three-point bending, and crush resistance/two plates test resulted in lower concentrations of stress on struts, thereby providing better structural integrity. Numerical results for Renuvia–PLLA stents showed that additional slots on the model for the radial compression test led to better effective stress distribution than in the case of the model without them, thus relaxing the structure of the stent regarding stress concentrations. On the other hand, for the inflation, three-point bending and crush resistance/two-plates test additional slots on the stent did not make any significant change on the effective stress distribution. These very encouraging conclusions testify that numerical simulations using our material model for bioresorbable PLLA stents can be used to imitate most of the standard *in vitro* tests for the evaluation of mechanical characteristics of the stents. Although we cannot claim that numerical simulations will fully replace demanding and expensive *in vitro* experiments, they have the potential to make a significant contribution to stent optimization and design processes, during almost all phases of stent development.

The results of our stress analysis can be used to determine appropriate design safety margins and to select the appropriate test articles (e.g., stent thickness or slot size) for durability testing (47). According to V& V 40 standard, question of interest of our computational modeling approach can be defined as: “Are the next-generation of BVS durable enough for the corresponding test?.” The context of use (the specific role and scope of the computational model used to address the QOI) may consist of the following steps:

1. A FE model will be used to simulate different tests: radial compression, inflation, three-point bending, and crush test, for stents with different strut thicknesses, and with different positions and sizes of pocket holes.
2. For each configuration of the proposed, next-generation bioresorbable vascular scaffold, the computational model will predict maximum principal stress at stent struts.
3. The worst-case *in vitro* prototype will be determined by the prototype with the highest predicted maximum principal stress.
4. The worst-case prototype will then be produced by the manufacturer and physically tested with the corresponding experimental machine.

All bioresorbable stent configurations must meet the test requirements for their intended use. Otherwise, as a consequence, device fracture can lead to restenosis, a serious disease condition, or even patient death. Since we suggest that the worst-case model should be physically tested on the experimental machine, the influence of the model is significant since simulation outputs from the computational model are a significant factor in the decision. Other details about step-by-step risk-informed credibility assessment were not in the scope of this study.

## Data Availability Statement

The datasets presented in this study can be found in online repositories. The names of the repository/repositories and accession number(s) can be found at: https://insilc.eu/.

## Author Contributions

MK and MM formulated and implemented the methodology. MM and DN designed the examples and analyzed the results. MA, VG, and BM performed the numerical simulations and prepared the results. NF provided the data and images for the reconstruction of the stent model examples and coordinated the project activities. All authors contributed to the article and approved the submitted version.

## Funding

This research is supported by the European Union's Horizon 2020 research and innovation programme under grant agreements No 777119 (https://insilc.eu/) and No 956470 (https://www.decodeitn.eu/). This article reflects only the author's view. The Commission is not responsible for any use that may be made of the information it contains. The research was also funded by Serbian Ministry of Education, Science, and Technological Development, grants [451-03-9/2021-14/200378 (Institute for Information Technologies, University of Kragujevac)] and [451-03-9/2021-14/200107 (Faculty of Engineering, University of Kragujevac)].

## Conflict of Interest

The authors declare that the research was conducted in the absence of any commercial or financial relationships that could be construed as a potential conflict of interest. The handling editor declared a shared consortium with the authors at time of review.

## Publisher's Note

All claims expressed in this article are solely those of the authors and do not necessarily represent those of their affiliated organizations, or those of the publisher, the editors and the reviewers. Any product that may be evaluated in this article, or claim that may be made by its manufacturer, is not guaranteed or endorsed by the publisher.
